# A Novel Capsule Endoscopic Score for Crohn’s Disease

**DOI:** 10.1093/crocol/otaa040

**Published:** 2020-05-17

**Authors:** Teppei Omori, Takayuki Matsumoto, Toshifumi Hara, Harutaka Kambayashi, Shun Murasugi, Ayumi Ito, Maria Yonezawa, Shinichi Nakamura, Katsutoshi Tokushige

**Affiliations:** 1 Institute of Gastroenterology, Tokyo Women’s Medical University, Tokyo, Japan; 2 Division of Gastroenterology, Department of Medicine, Iwate Medical University, Morioka, Japan

**Keywords:** Crohn’s Disease Activity in Capsule Endoscopy, Lewis Score, Capsule Endoscopy Crohn’s Disease Activity Index, Crohn’s disease, capsule endoscopy

## Abstract

**Background and Aims:**

The Lewis Score (LS) and Capsule Endoscopy Crohn’s Disease Activity Index (CECDAI) are the two currently used small bowel capsule endoscopy (SBCE) scoring systems for Crohn’s disease (CD). The present study describes a new scoring system for evaluation of small bowel CD, especially mucosal inflammation.

**Methods:**

In this cross-sectional study, 108 CD patients underwent 196 SBCEs. The small bowel lesions were scored using our new Crohn’s Disease Activity in Capsule Endoscopy (CDACE). CDACE is the sum of scores for location of inflammation, range of inflammation, and stenosis, with a value ranging from 0 to 1643. We analyzed the relation between CDACE and LS, CECDAI, CDAI, and CRP values and evaluated the inter-rater reliability of CDACE using the intraclass correlation coefficient (ICC) (2.1).

**Results:**

The mean (±SD) values of LS, CECDAI, and CDACE were 501 ± 1177, 5.8 ± 5.4 and 431 ± 356, respectively. CDACE correlated significantly with LS and CECDAI (*ρ* = 0.737, *P* < 0.0001 for LS and *ρ* = 0.915, *P* < 0.0001 for CECDAI). CDACE also correlated significantly with CDAI (*ρ* = 0.36) and CRP (*ρ* = 0.23). The ICC (2.1) was 0.829, indicating strong agreement among readers.

**Conclusions:**

CDACE is a potentially useful SBCE scoring system for small bowel CD, as it represents the extent and spread of small bowel mucosal inflammation and stenosis.

## INTRODUCTION

Small bowel capsule endoscopy (SBCE) is a minimally invasive procedure used to visualize inflammatory changes in the small bowel mucosa of Crohn’s disease (CD). Several professional guidelines have emphasized the clinical usefulness of SBCE in CD.^1–7^ At present, small bowel CD lesions detected by SBCE are assessed using 2 scoring systems: the Lewis Score (LS)^8^ and the Capsule Endoscopy Crohn’s Disease Activity Index (CECDAI).^9^ The LS evaluates the inflammatory changes in the small bowel mucosa using the parameters of villous edema, ulceration, and stenosis. For quantitative evaluation, the small bowel capsule transit time is divided into tertiles. For each quartile, the number, extent, and size of the villous edema and ulceration are multiplied, and the largest quartile value is then added to an independent stenosis score. In the CECDAI, the small intestine is divided into proximal and distal segments based on the small bowel transit time. Each section is evaluated for inflammation, extent, and stenosis and the sum of these scores is calculated.

While several reports examined the correlation among these 2 scoring systems,^10–12^ CD presents with a wide variety of pathologies, including inflammation and stenosis, making it impossible to assess the pathology accurately using these scores alone. Because the LS and CECDAI are derived from the sum of individual scores, they are probably inappropriate for the assessment of disease pathology. Small bowel lesions in CD patients identified by the SBCE are inflammatory changes, whereas the stenotic lesions are either inflammatory (edematous) stenosis or mild organic constriction, through which the patency capsule (PC) passes. Based on these pathological features, we considered that assignment of different coefficients weights that differentiate inflammation from stenotic changes can provide a score that better reflects the disease status, especially mucosal inflammation.

In this report, we describe our new capsule endoscopic scoring system designed for easier assessment of the pathology and severity of CD.

## MATERIALS AND METHODS

SBCE was conducted in patients with an established diagnosis of CD affecting the small intestine, who had been evaluated previously for patency of the small intestine with a PC. The SBCE examination described in this study was conducted as part of the clinical assessment of the patients to evaluate the status of small bowel lesions. This retrospective study included 196 SBCE sessions performed in 108 CD patients at our hospital between June 2010 and June 2018. Patients were excluded if (1) they exhibited obvious intestinal stenosis or obstruction depicted with other examinations that would likely make capsule passage impossible, (2) they were not more than 15 years of age; or (3) intestinal patency had not been confirmed by a PC (Medtronic, Minneapolis, MN) (Table 1).

**Table 1.** Demographic and Clinical Characteristics of the Patients

SBCE sessions, n	196
SBCE sessions (males/females), n	131/65
Patients (males/females), n	108 (69/39)
Age, years	37.6 ± 15.3
Disease duration, months	105 ± 100
Hemoglobin (g/dL)	13.2 ± 2.0
Platelet count (10^4^/μL)	26.3 ± 9.6
Albumin (mg/dL)	4.1 ± 0.6
C-reactive protein (mg/dL)	0.74 ± 1.5
CDAI	107 ± 85
Classification of Montreal	
A1	37 (18.9)
A2	122 (62.2)
A3	37 (18.9)
L1	65 (33.2)
L2	0 (0)
L3	131 (66.8)
B1	95 (48.5)
B2	63 (32.1)
B3	38 (19.4)
Perianal disease	50 (25.5)
Past intestinal surgery	85 (43.4)
Smoking	26 (13.3)
Medications	
5ASA	162 (82.7)
Steroid	26 (13.3)
Azathioprine	36 (18.4)
Elemental diet (300–600 kcal/day)	101 (51.5)
Anti-TNFα (infliximab/adalimumab)	92/27 (60.7)
LS	501 ± 1177 [0–5701]
CECDAI	5.8 ± 5.4 [0–25]
SB2/SB3	75/121

Data are mean ± SD or number (%) [range] of patients

The study protocol was approved by the Human Ethics Review Committee of our university and each patient provided written informed consent. This study was registered in the Clinical Trials Registry, July 27, 2018 (4816R).

We investigated whether inflammation in various segments of the intestine and existence of stenosis could be inferred from the score values of the 3 scoring systems: LS, CECDAI, and our novel scoring system, the Crohn’s Disease Activity in Capsule Endoscopy (CDACE) (see SBCE Scoring Systems). While the definitions of the ranges are different for each scoring system, we selected the following definitions of “multilevel bowel lesion.” In LS, detection of inflammation in more than ⅔ of the small intestine. In CECDAI, presence of inflammation in both the proximal and distal segments of the small intestine. In CDACE, inflammation in more than one segment of the intestine, excluding 2 consecutive zones. Because CDACE divides the small bowel into 4 quartiles, 2 consecutive zones may correspond to one range of other scores. Therefore, the “multilevel bowel lesion” of CDACE was defined excluding 2 consecutive zones.

We also evaluated the interrater reliability of CDACE using the intraclass correlation coefficient (ICC) (2.1). Twenty patients forming a representative CDACE score range after anonymization and randomization were included in the analysis (CDAI: 168 ± 115, LS: 566 ± 1191; range: 0–3961, CECDAI: 6.6 ± 4.4; range: 0–13). Two gastroenterologists (T.H., H.K.) with more than 50 SBCEs experience independently interpreted the image records and determined the LS, CECDAI, and CDACE scores. We analyzed the agreement in the scores between these 2 gastroenterologists as well as with those by practitioners with experience of more than 1000 SBCEs (obtained from the medical records).

### SBCE Procedure

Intestinal patency was confirmed in all cases by PC (Medtronic) before the SBCE procedure. SBCE was performed by practitioners with experience of more than 1000 SBCEs. The capsule endoscopy was the PillCam SB2 or SB3 (Medtronic).

The patient was instructed to fast from 9:00 pm on the day before SBCE examination. At 8:00 am on the following morning, 15 mg mosapride was administered orally as a pretreatment drug. The intestinal cleansing procedure was considered unnecessary for SBCE. The time of swallowing the SBCE with water marked the start of the SBCE procedure. Drinking water was provided 2 hours after ingestion, and meal 4 hours after swallowing the SBCE. The excretion of the capsule was confirmed visually after completion of the examination.

### SBCE Scoring Systems

Our novel scoring system, the CDACE, was established with particular emphasis on the evaluation of mucosal inflammation and the presence of stenotic lesions. In this study, we also correlated the CDACE score with those of the LS and CECDAI.

**Figure 1.**
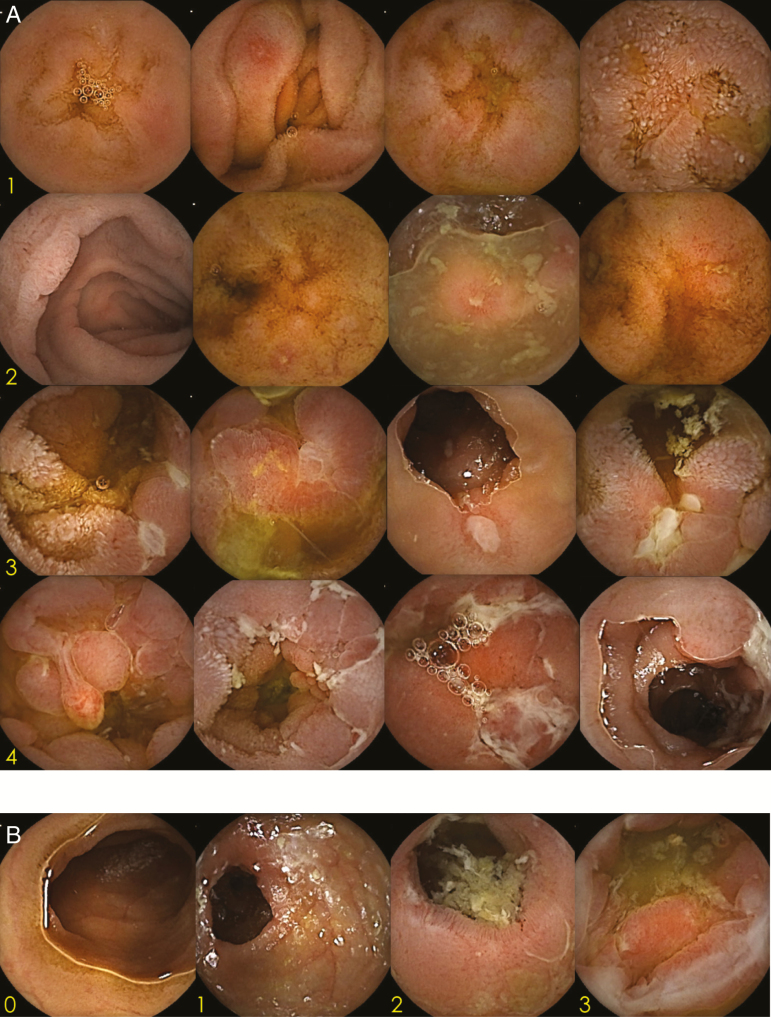
A, CDACE Li score. The severity of inflammation was assessed in each quartile using the following grading system: 0, normal mucosa; 1, edematous/reddish; 2, erosion (size, <0.5 cm); 3, irregular/circular ulcer (size, 0.5–2 cm); and 4, longitudinal/large ulcer/cobblestone appearance. The scores of the 4 quartiles were then summed up to obtain the Li score. Theoretically, the Li score ranges from 0 to 16. Li, location inflammation; CDACE, Crohn’s Disease Activity in Capsule Endoscopy. B, CDACE S score. The presence or absence of stenosis in the entire small intestine was evaluated by using 4 grades to obtain the S score: 0, no stenosis; 1, single passage; 2, multiple passages; and 3, no passage. Theoretically, the S score ranges from 0 to 3. S, stenosis.

Using the progress indicator function of the software, thumbnails are created at 25%, 50%, and 75% of the SBCE images of the small bowel (Supplementary Fig. S1). The thumbnails can be captured in the blue mode, or by using other methods, for easy recognition. After dividing the small bowel into 4 quartiles, the severity of inflammation is assessed in each quartile using one of 5 grades: 0, normal mucosa; 1, edematous/reddish; 2, erosion (size, <0.5 cm); 3, irregular/circular ulcer (size, 0.5–2 cm); and 4, longitudinal/large ulcer/cobblestone appearance. The scores of the 4 quartiles are then summed up to obtain the location inflammatory (Li) score (range: 0–16). Since the Li score is what makes the CDACE specific to CD, high scores are given to longitudinal ulcers, ulcers with cobblestone appearance, and extensive ulcers (Fig. 1A). The small bowel quartiles encompassing the inflammatory lesions were counted to obtain the range (R) score (0–4). In addition to the Li and R scores that describe small bowel inflammation, the CDACE also incorporates the stenosis (S) score, which denotes the presence or absence of stenosis throughout the entire small intestine. The S score (range: 0–3) is based on a 4-grade system: 0, no stenosis; 1, single passage; 2, multiple passages; and 3, no passage (Fig. 1B). When the examination is completed without the SBCE reaching the colon, the small bowel is scored by dividing the small bowel into 4 quartiles with the last image considered the end segment of the small bowel.

The CDACE score is calculated using the following formula: Li score × 100 + R score × 10 + S score and ranges from 0 to 1643 (Table 2). The digits of the CDACE score provide a visual reading of the status of the small bowel inflammation. Thus, from the left, the first two digits of the score denote the location of inflammation in the small intestine, the third digit defines the extent of small bowel inflammation, and the last fourth digit denotes the presence or absence of stenosis. Furthermore, the first and second digits divided by the third digit represents the severity of inflammation. For example, a CDACE score of 0620 indicates Li of 06 (digits 06, ie, 600 in CDACE score), inflammation limited to 2 of the 4 small bowel quartiles (third digit 2), no stenosis (last digit, 0), and severity of inflammation of 3 (6/2). On the other hand, a CDACE score of 1643 indicates Li of 16, extensive inflammation throughout all the 4 small bowel segments, with 3 no passage (with stenosis).

**Table 2.** Outline and Calculation of CDACE

Location inflammatory score	Inflammatory score of each location (Li1, Li2, Li3, Li4)
0	Normal mucosa
1	Edematous/reddish
2	Erosion (<0.5 cm)
3	Irregular/circular ulcer (0.5–2 cm)
4	Longitudinal/large ulcer/cobblestone appearance
Range score	R score
0	No inflammation
1	Inflammation within 1/4 quartile
2	Inflammation within 2/4 quartiles
3	Inflammation within 3/4 quartiles
4	Inflammation within 4/4 quartiles
Stenosis score	S score
0	No stenosis
1	Single passage
2	Multiple passages
3	No passage

CDACE score = (Li1 + Li2 + Li3 + Li4) × 100 + R score × 10 + S score; score range: 0–1643.

The LS was calculated using the SBCE interpretation software Rapid 8.3 (Medtronic), while the CECDAI was calculated manually.

### Statistical Analysis

All data were expressed as mean ± standard deviation. Spearman’s rank correlation coefficient was used to analyze the correlations of the CDACE score with those of the LS, CECDAI, CDAI, and CRP. *P* values of less than 0.05 were considered significant. With regard to the presence or absence of inflammation on multiple digits and the presence or absence of stenosis, the cutoff values for each score was calculated using the AUC obtained from the ROC curve, and the sensitivity, specificity, positive predictive value (PPV), and negative predictive value (NPV) were calculated. We also evaluated the interrater reliability for CDACE, using the ICC (2.1). The Shapiro–Wilk W-test was used to test the normality of the extracted CDACE data.

## RESULTS

### Characteristics of Patients

The subjects included 131 men and 65 women with a mean age of 37.6 ± 15.3 years. The mean CD duration was 105 ± 100 months. Table 1 presents the clinical characteristics, including the numbers of SBCE sessions under Montreal classifications and medications used at the time of SBCE. The mean CDAI was 107 ± 85, while the mean CRP was 0.74 ± 1.5 mg/dL. The scores of the LS and CECDAI were 501 ± 1177 (range 0–5701) and 5.8 ± 5.4 (0–25), respectively (Table 1), and the 2 scores showed a significant correlation by Spearman’s rank correlation analysis (*ρ* = 0.816, *P* < 0.0001) (Supplementary Fig. S2).

### Correlation Between CDACE and SBCE Scoring System

**Figure 2.**
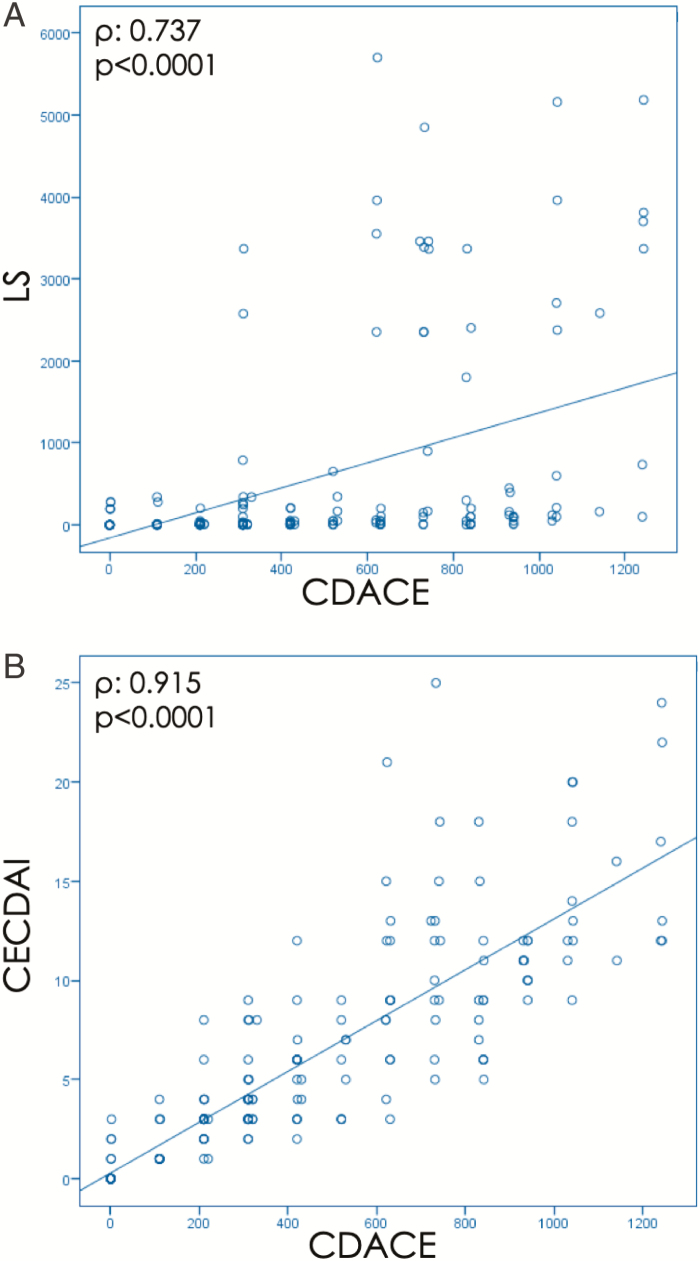
A, Correlation between CDACE and LS. Correlation between CDACE and LS was *ρ* = 0.737 (*P* < 0.0001). CDACE, Crohn’s Disease Activity in Capsule Endoscopy; LS, Lewis Score. B, Correlation between CDACE and CECDAI. Correlation between CDACE and CECDAI was *ρ* = 0.915 (*P* < 0.0001). CECDAI, Capsule Endoscopy Crohn’s Disease Activity Index.

**Figure 3.**
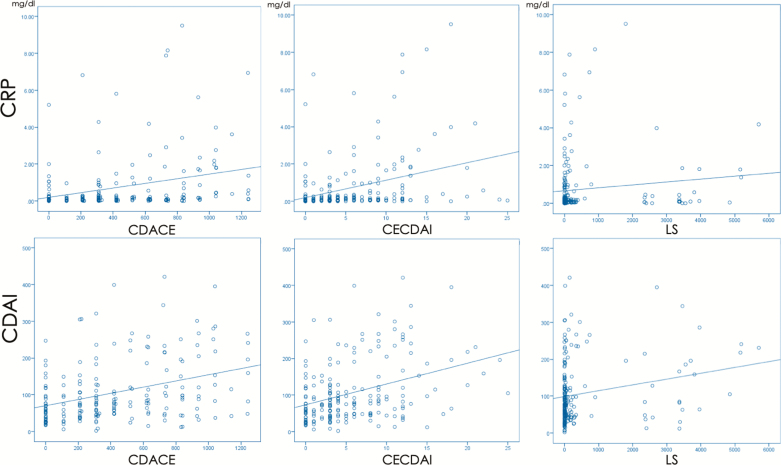
Correlation of CDACE, CECDAI, and LS with CDAI and CRP. Correlations of CDACE, LS, and CECDAI with CDAI (*ρ* = 0.346, *P* < 0.0001; *ρ* = 0.246, *P* = 0.001; and *ρ* = 0.347, *P* < 0.0001, respectively) and CRP (*ρ* = 0.355, *P* < 0.0001; *ρ* = 0.232, *P* = 0.001; and *ρ* = 0.327, *P* < 0.0001, respectively). CDACE, Crohn’s Disease Activity in Capsule Endoscopy; CECDAI, Capsule Endoscopy Crohn’s Disease Activity Index; LS, Lewis Score; CDAI, Crohn’s Disease Activity Index; CRP, C-reactive protein.

The CDACE score was 431 ± 356 (range 0–1243) and correlated strongly and significantly with those of LS (*ρ* = 0.737, *P* < 0.0001; Fig. 2A) and CECDAI (*ρ* = 0.915, *P* < 0.0001; Fig. 2B). The correlations of the CDACE, LS, and CECDAI with the CDAI were *ρ* = 0.346 (*P* < 0.0001), *ρ* = 0.246 (*P* = 0.001), and *ρ* = 0.347 (*P* < 0.0001), respectively, and those with the CRP were *ρ* = 0.355 (*P* < 0.0001), *ρ* = 0.232 (*P* = 0.001), and *ρ* = 0.327 (*P* < 0.0001), respectively (Fig. 3). Each capsule endoscopy score showed a weak correlation with CDAI and CRP.

For LS, the score cutoff value was 9 at the maximum value of 0.851 of the AUC of the ROC curve. Using this cutoff value, the sensitivity of LS for the presence of inflammation (more than ⅔) was 84%, with 74% specificity, 76% PPV, and 82% NPV. For CECDAI, at AUC of 0.958 (maximum value on the ROC curve), the cutoff score was 4.5. Using this cutoff value, the sensitivity of the CECDAI for the presence of inflammation (both the proximal and distal bowel) was 94%, specificity 86%, PPV 83%, and NPV 95%. For CDACE, the cutoff value was 316 at the maximum value of 0.977 on the AUC of the ROC curve. With this value, the sensitivity for the presence of inflammation at multiple sites (excluding 2 adjacent zones) was 100%, specificity 82%, PPV 79%, and NPV 100%.

**Figure 4.**
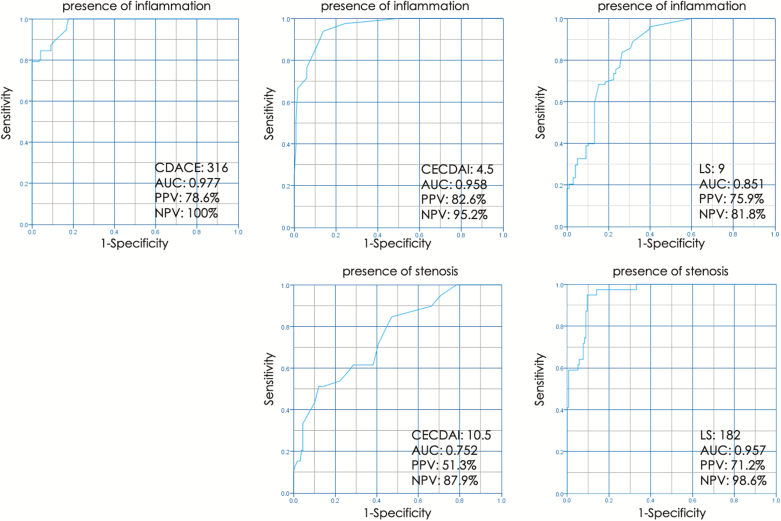
Sensitivity, specificity, PPV, and NPV. With regard to the presence or absence of inflammation on multiple digits and the presence or absence of stenosis, the cutoff value of each score was calculated from the ROC curve, and the sensitivity, specificity, PPV, and NPV were computed. PPV, positive predictive value; NPV, negative predictive value.

With regard to bowel stenosis, the LS score cutoff value was 182 at the maximum value of AUC of the ROC of 0.957. Using this cutoff value, the sensitivity of LS in detecting bowel stenosis was 95%, with the specificity of 90%, PPV of 71%, and NPV of 99%. For CECDAI, at the maximum value of AUC of the ROC curve of 0.752, the CECDAI score cutoff was 10.5. Using this cutoff value, the sensitivity of detection of stenosis was 51%, specificity 88%, PPV 51%, and NPV 88%. For CDACE, the fourth digit score of CDACE (ie, last digit from the left) reflects the extent of stenosis in the intestine (Fig. 4).

### Evaluation of Interrater Reliability of CDACE

Analysis of data of 20 patients with a representative CDACE score range after anonymization and randomization showed no significant differences in the tests of normality (Shapiro–Wilk W-test: *P* = 0.304) (Supplementary Table S1 and Fig. S3). Table 3 presents the validity and rate of agreement among the readers. The CDACE correlated with the original score and strongly with CECDAI and LS (Supplementary Table S2). Among the 3 readers, the ICC (2.1) of CDACE was 0.829 (95% confidence interval: 0.630–0.928), indicating a strong agreement (Table 3).

**Table 3.** Validity and Rate of Agreement Among Readers on the CDACE

	CDACE	ICC (2.1)	95% CI
Expert	594 ± 395 [0–1243]		
Reader, T.H.	760 ± 351 [110–1342]	0.829	0.630–0.928
Reader, H.K.	546 ± 357 [0–1340]		

## DISCUSSION

Two indexes are commonly used in the endoscopic evaluation of the CD, including the Crohn’s Disease Endoscopic Index of Severity^13^ and Simple Endoscopic Score for Crohn’s Disease.^14^ Furthermore, the Rutgeerts score^15^ is used for evaluation of patients who had undergone intestinal resection. However, even with the use of these scoring systems, it is often difficult to evaluate small bowel regions. On the other hand, the CDAI and CRP do not describe small bowel lesions.^16–18^ Thus, the SBCE procedure seems the most suitable system that provides detailed information on small bowel status in CD.^1–7^ Currently, the LS^8^ and CECDAI^9^ are used to assess the severity of small bowel CD lesions detected by the SBCE. Both systems have been validated extensively^19, 20^ and recommended by various professional societies.^5, 6^ However, both the LS and CECDAI have several limitations. In this study, the sensitivity and specificity of the LS in detecting bowel inflammation of (⅔ or more) were 84% and 74% (using a cutoff value of 9), respectively. On the other hand, the sensitivity and specificity of detecting bowel stenosis by the LS were 95% and 90%, respectively, using a cutoff value of 182. In comparison, the respective values for CECDAI were 94% and 86% (using cutoff value of 4.5), for inflammation, and 51% and 88%, with a cutoff value of 10.5, for bowel stenosis. With regard to LS, even in cases with inflammation affecting the entire small bowel, the score represents one of the 3 segments of the small bowel. In other words, the severity of inflammation is evaluated with LS without taking the extent of bowel inflammation into consideration. Second, the LS score can be high even in cases with mucosal healing, because the score increases with the presence of stenosis.^21^ Therefore, setting the cutoff value to 182 probably increased the sensitivity and specificity of LS for the diagnosis of stenosis.

The CECDAI divides the small bowel into 2 segments based on the transit time, and the score is the sum of the inflammatory score and stenosis score. Thus, the CECDAI provides a better assessment of inflammation throughout the small bowel compared with the LS. We believe that setting the cutoff value of CECDAI at 4.5 increased the sensitivity and specificity for the identification of cases with inflammation affecting the entire small intestine. On the other hand, in CECDAI, the stenosis score is less likely to affect the total score. This may explain why it can be difficult to judge the presence or absence of stenosis from the CECDAI score value. Since the LS and CECDAI are derived from the sum of individual scores, they are probably inappropriate for the assessment of disease pathology.

We designed the CDACE to simplify the assessment of the extent of bowel inflammation, reduce the impact of stenosis on the total score, and allow easy visual estimation of the severity of small bowel lesions from the value of the CDACE score. The CDACE comprises the Li score, R score, and S score. For the Li and S scores, we implemented, in part, the preexisting systems. For the R score, we divided the small bowel into 4 parts by the progress indicator. The numbers of quartiles with positive findings were counted to obtain the R score. The location of the digits in the CDACE provides information that can be used to interpret the state of inflammation; the third digit reflects the extent of small bowel inflammation. The severity of inflammation can be inferred by dividing the first and second digits by the third digit (range of inflammation). Furthermore, the fourth digit reflects the severity of stenosis. Thus, the CDACE provides a numeric image of the overall pathology of the small intestine.

In CDACE, the presence or absence of stenosis has almost no influence on the total score, its status is reflected by the fourth digit of the score. The CDACE focused more on inflammatory findings than other scores. Therefore, in the CDACE, the stenosis score is set low. For Crohn’s disease, stenotic lesions are a serious prognostic complication. However, unlike treatment for inflammation, treatment for fibrous stenosis is surgical or endoscopic balloon dilation.^22–24^ For this reason, the score for evaluating CD must consider the stenosis and inflammation factors separately. By placing the element of stenosis as the fourth digit of the score, CDACE clarified the extent of inflammation. In other words, a stenosis score in the fourth digit is not recognized as an inflammation score. This may make it possible to determine the effect of treatment on inflammation and to compare the severity of inflammation. We believe that setting the stenosis score as low as the fourth digit does not mean that we are underestimating the stenosis. Moreover, it is possible to assess the presence of stenosis by considering the score only and thus can be used to identify patients in whom SBCE passage is impossible and who would require interventions against the stenosis. Importantly, should the examination be completed without the SBCE reaching the colon, our scoring system cannot evaluate inflammation of the whole small bowel, as with the other scoring systems. However, since the stenosis score in such cases is 3, it should clearly indicate that the whole small bowel cannot be observed. Even in such cases, magnetic resonance enterography or balloon-assisted enteroscopy is recommended to accurately assess the small bowel from the CDACE score results. Table 4 presents the advantages and disadvantages of the LS, CECDAI, and CDACE.

**Table 4.** Advantages and Disadvantages of the Lewis Score, CECDSI, and CDACE on the Basis of Our Study Results

	Lewis Score	CECDAI	CDACE
Advantage	• Automatic calculation by software. • Easy recognition of stenosis (with high scores).^21^ • With validation.^19^	• Reflects the degree of inflammation in the entire small intestine compared to LS.^21^ • With validation.^20^	• It shows extent of inflammation. • Provide information on presence and extent of stenosis. • Correlates with existing scores.
Disadvantage	• Only one third of the most inflamed small intestine areas are reflected in the score. • Are high scores due to inflammation or stenosis?^21^	• Manual calculation. • No clue to presence or absence of stenosis from the score values.^21^	• Manual calculation. • Without validation (this manuscript only).

Supplementary Figure S4A and B shows examples of how the CDACE score allows easy assessment of small intestine pathology. A CDACE of 0210 indicates that the severity of the inflammation involves erosion (digits 02), inflammation in one of the 4 small bowel quartiles (digit 1), and no stenosis (digit 0) (Supplementary Fig. S4A). On the other hand, a CDACE score of 1241 indicates that inflammation is present throughout the small bowel with one stenotic site. While this exists in various forms of severity, in general, it can be interpreted as longitudinal ulcers in 2 of the 4 quartiles (50%) or ulcers of 0.5–2.0 cm scattered throughout the intestine (Supplementary Fig. S4B). For accurate mapping of intestinal inflammation, the formula used to calculate the CDACE should also be shown. Our results also showed a significant correlation between CDACE and the LS and CECDAI. In addition, strong agreement on CDACE scores was noted among the different readers. The correlation with the CECDAI was particularly strong, probably due to the fact that the stenosis score has a minimal impact on the score of each system. In addition, CDACE matches the existing scoring systems with regard to its correlation with CRP and CDAI.

This study has several limitations. First, the CDACE cannot be used to assess CD with severe stenosis in which the PC does not pass through. Second, in this study, we used both the SB2 and SB3 capsules. The SB3 capsule is equipped with an AFR (Adaptive Frame Rate) system with a frame rate of 2 or 6 frames per second. This means that the SB3 capsules recognize intestinal lesions more likely than the SB2 capsule. Despite this difference, the CDACE score does not take into account the number of lesions per segment, but rather uses characteristic features as the inflammation score. Therefore, the CDACE score is not affected by the AFR. Furthermore, the analysis used retrospective data. In addition, the inclusion of L3 of Montreal classification may have influenced the correlation between CDACE and CRP or CDAI. Furthermore, it is difficult to confirm the power of the CDACE in the assessment of severity based on this study alone.

In conclusion, CDACE may be a potentially useful SBCE scoring system for small bowel CD, as it represents the extent and spread of small bowel mucosal inflammation and stenosis. Further studies are needed to validate the results described here and assess the clinical utility of the CDACE score.

## Supplementary Material

otaa040_suppl_Supplementary_Figure_S1

otaa040_suppl_Supplementary_Figure_S2

otaa040_suppl_Supplementary_Figure_S3

otaa040_suppl_Supplementary_Figure_S4a

otaa040_suppl_Supplementary_Figure_S4b

otaa040_suppl_Supplementary_Figure_Legend_and_Tables

## Data Availability

Data are available at doi:10.5281/zenodo.3765953.

## Notes

Funding: This research work was conducted without financial support from any public, private, or not-for-profit funding agency.

Author Contribution: T.O.: Study concept and design; H.K., S.M., A.I., M.Y., and T.O.: Data acquisition; T.O.: Statistical analysis and drafting of the first version of the manuscript; T.O., S.N., K.T., and T.M.: Critical revision and approval of the final version of the manuscript.
