# Supplementary figures and images for: A Novel Capsule Endoscopic Score for Crohn’s Disease
Source: Crohns Colitis 360. 2020 May 17;2(2):otaa040. doi: 10.1093/crocol/otaa040 (PMC9802418; doi:10.1093/crocol/otaa040)

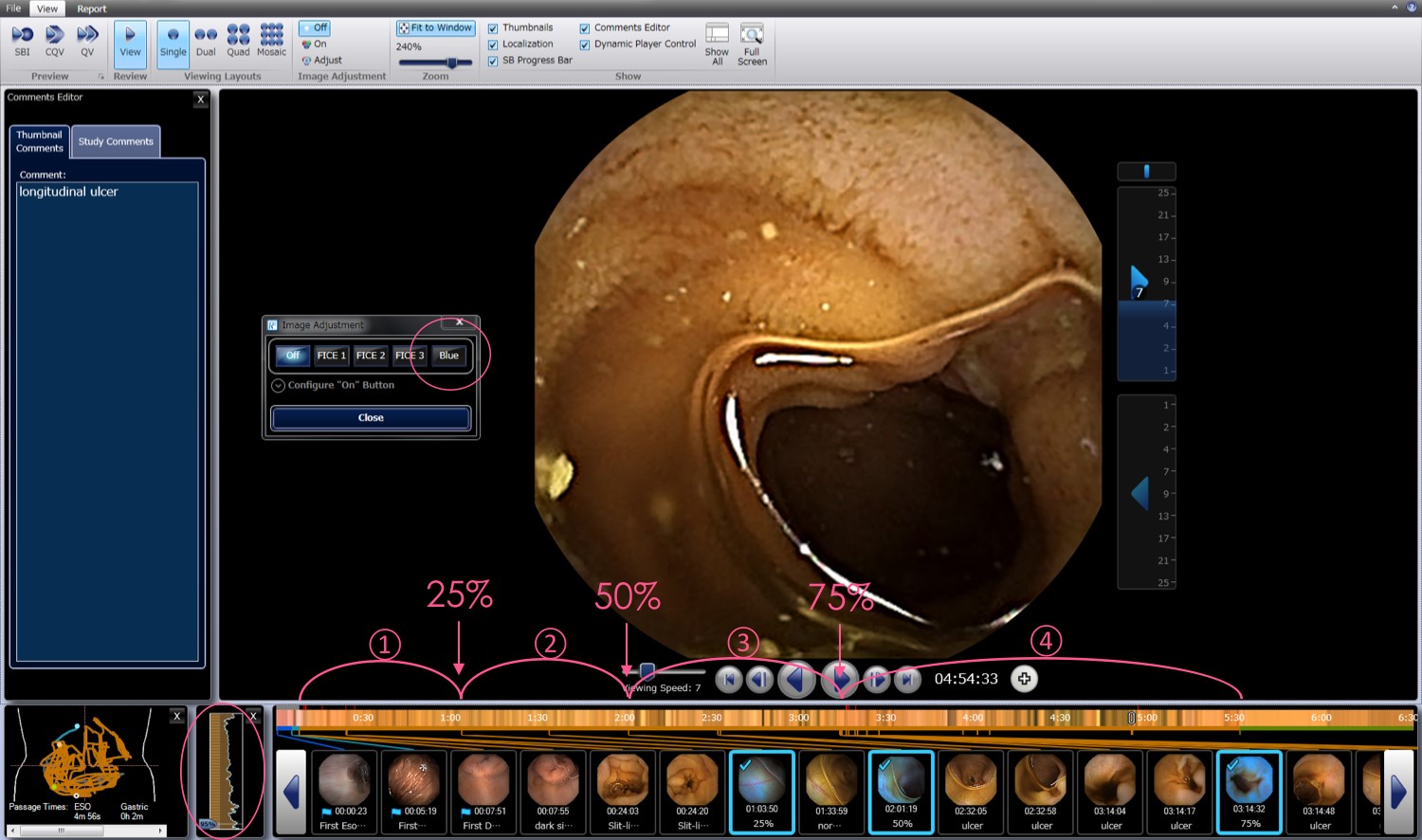

Supplement: otaa040_suppl_Supplementary_Figure_S1 [file otaa040_suppl_supplementary_figure_s1.jpeg]

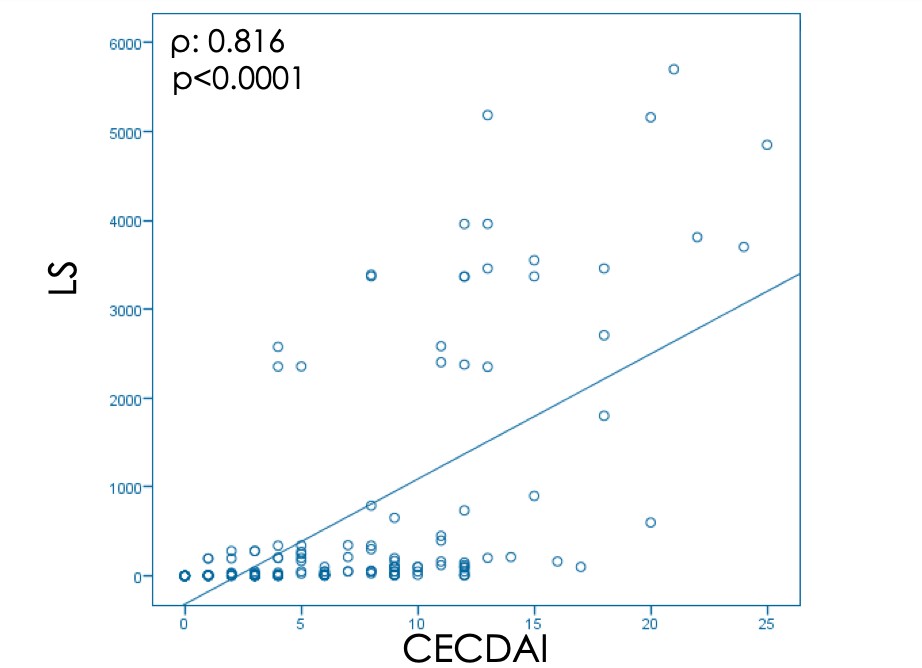

Supplement: otaa040_suppl_Supplementary_Figure_S2 [file otaa040_suppl_supplementary_figure_s2.jpeg]

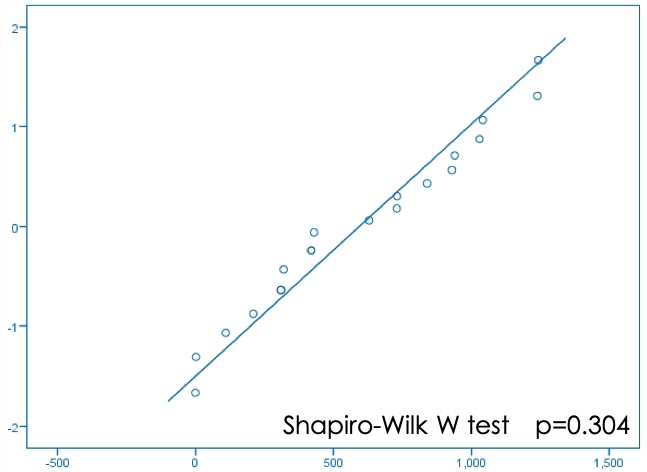

Supplement: otaa040_suppl_Supplementary_Figure_S3 [file otaa040_suppl_supplementary_figure_s3.jpeg]

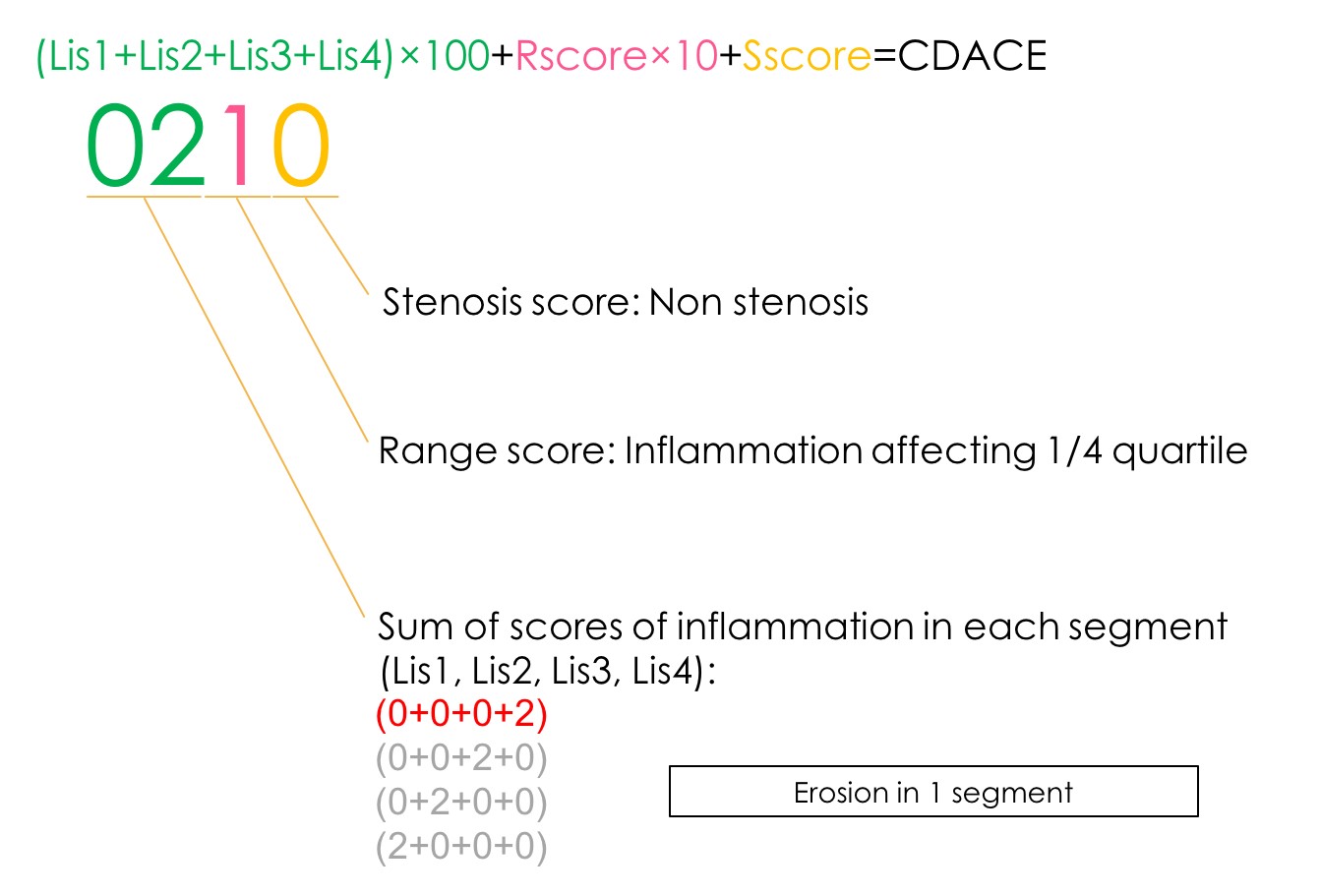

Supplement: otaa040_suppl_Supplementary_Figure_S4a [file otaa040_suppl_supplementary_figure_s4a.jpeg]

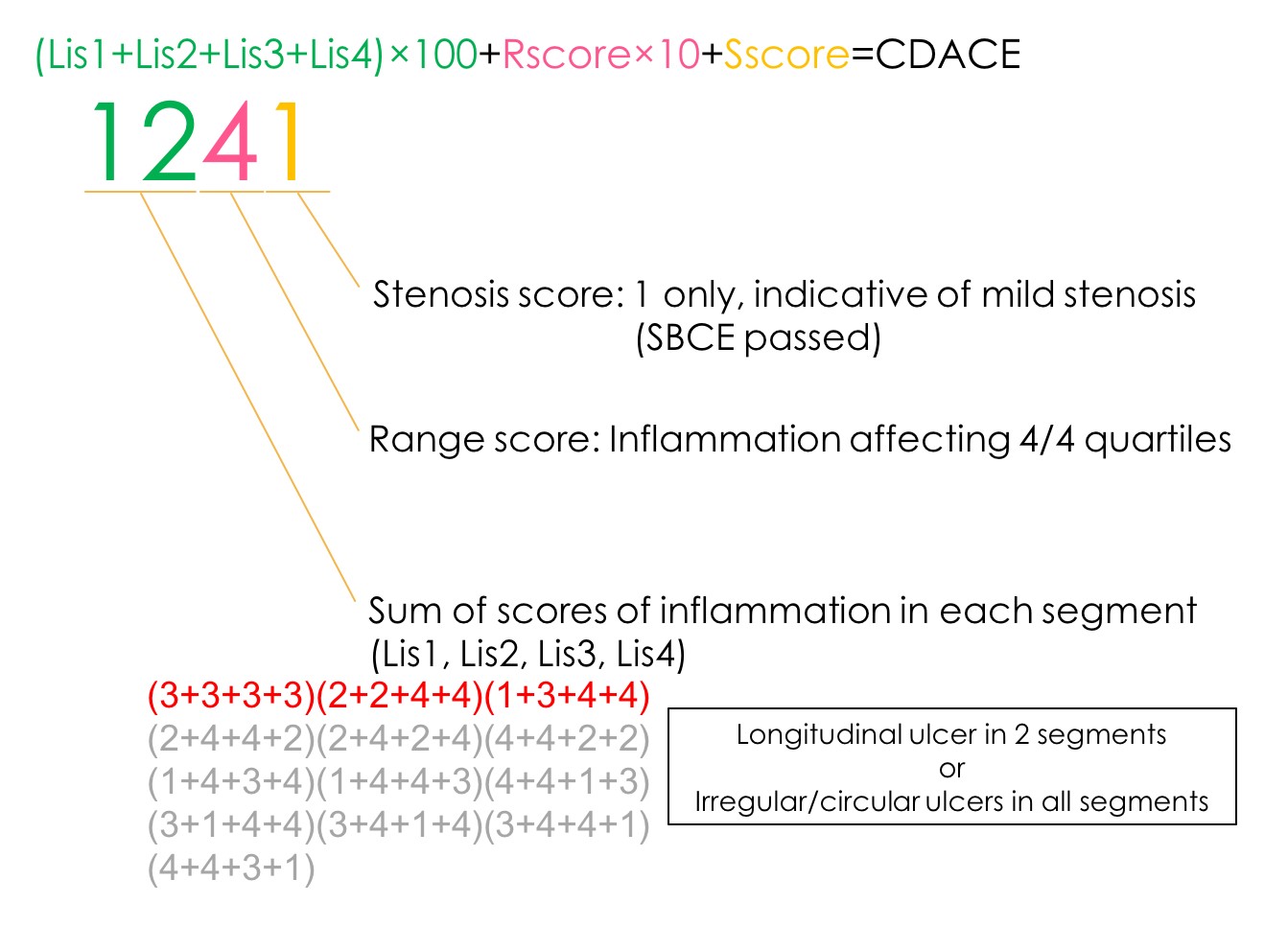

Supplement: otaa040_suppl_Supplementary_Figure_S4b [file otaa040_suppl_supplementary_figure_s4b.jpeg]
