# Supplementary material for: A Novel Capsule Endoscopic Score for Crohn’s Disease
Source: Crohns Colitis 360. 2020 May 17;2(2):otaa040. doi: 10.1093/crocol/otaa040 (PMC9802418; doi:10.1093/crocol/otaa040)
Supplement: otaa040_suppl_Supplementary_Figure_Legend_and_Tables [file otaa040_suppl_supplementary_figure_legend_and_tables.docx]

**Supplementary Material**

**Figure S1. Crohn’s Disease Activity in Capsule Endoscopy settings.** The progress indicator function of the interpretation software (Rapid 8.3, Medtronic, Minneapolis, MN) was used to create thumbnails at 25%, 50%, and 75% along the observed small bowel. The thumbnails can be captured in the blue mode or using another method for easy recognition. The three thumbnails divide the small bowel into four quartiles.

**Figure S2. Correlation between LS and CECDAI.** The mean LS was 501±1177 (range, 0-5701), while the mean CECDAI was 5.8±5.4 (0-25). Spearman’s rank correlation analysis showed a strong correlation between the LS and CECDAI scores (ρ=0.816, p<0.0001). LS: Lewis Score; CECDAI: Capsule Endoscopy Crohn’s Disease Activity Index**.**

**Figure S3. Normality of the extracted CDACE data.** In 20 patients with representative CDACE score range after anonymization and randomization, there were no significant differences in the tests of normality (Shapiro-Wilk W test: p=0.3042). CDACE: Crohn’s Disease Activity in Capsule Endoscopy.

**Figure S4. (a) CDACE example 1.** A CDACE of 0210 indicates that the severity of the inflammation involves erosion (first two digits from the left, 02), inflammation limited to one of the four small bowel quartiles (third digit 1), and no stenosis (fourth digit 0). CDACE: Crohn’s Disease Activity in Capsule Endoscopy. (**b) CDACE example 2.** A CDACE of 1241 indicates the presence of inflammation throughout the small bowel (first two digits from the left, 12 out of a maximum of 16, plus the third digit of 4) plus a single stenosis (fourth digit, 1). While the severity of the latter varied across the intestine, the general image is of longitudinal ulcers in two of the four quartiles (50%) or ulcers of 0.5-2.0 cm scattered throughout. CDACE: Crohn’s Disease Activity in Capsule Endoscopy.

**Table S1.** Twenty patients with representative CDACE score range after anonymization and randomization.

| CDACE calculation | CDACE | CECDAI calculation | CECDAI | Lewis score |
| --- | --- | --- | --- | --- |
| (0+0+0+0)00 | 0 | （0×0+0）（0×0+0） | 0 | 0 |
| (0+0+0+0)02 | 2 | （0×0+2）（0×0+1） | 3 | 280 |
| (0+0+0+1)10 | 110 | （0×0+0）（1×1+0） | 1 | 1 |
| (0+0+0+2)10 | 210 | （0×0+0）（3×1+0） | 3 | 25 |
| (0+0+0+3)10 | 310 | （0×0+0）（3×1+0） | 3 | 6 |
| (0+0+3+0)10 | 310 | （0×0+0）（2×1+0） | 2 | 3 |
| (0+2+0+1)20 | 320 | （3×1+0）（1×1+0） | 4 | 8 |
| (0+0+1+3)20 | 420 | （0×0+0）（3×1+0） | 3 | 12 |
| (0+0+2+2)20 | 420 | （0×0+0）（3×2+0） | 6 | 18 |
| (0+1+2+1)30 | 430 | （3×1+0）（1×1+0） | 4 | 6 |
| (0+2+1+3)30 | 630 | （3×1+0）（3×1+0） | 6 | 6 |
| (2+0+2+3)30 | 730 | （3×1+0）（3×2+0） | 9 | 10 |
| (0+2+2+3)31 | 731 | （3×2+0）（3×2+1） | 13 | 2352 |
| (2+2+2+2)40 | 840 | （3×1+0）（3×1+0） | 6 | 18 |
| (2+0+3+4)30 | 930 | （3×1+0）（4×2+0） | 11 | 450 |
| (2+2+2+3)40 | 940 | （3×1+0）（3×2+0） | 9 | 10 |
| (0+4+4+2)30 | 1030 | （4×2+0）（4×1+0） | 12 | 50 |
| (2+2+4+2)42 | 1042 | （3×1+0）（4×2+2） | 13 | 3961 |
| (2+4+4+2)40 | 1240 | （4×2+0）（4×1+0） | 12 | 736 |
| (3+3+3+3)43 | 1243 | （3×1+3）（3×1+3） | 12 | 3370 |

**Table S2.** Correlation of CDACE with the existing scores.

| n=20 | LS | CECDAI | CDACE | Spearman‘s rank correlation coefficient | | |
| --- | --- | --- | --- | --- | --- | --- |
|  |  |  |  | LS vs.  CECDAI | LS vs.  CDACE | CECDAI vs. CDACE |
| Expert | 566±1192  [0-3961] | 6.6±4.4  [0-13] | 594±395  [0-1243] | ρ=0.784, p<0.0001 | ρ=0.662, p<0.0001 | ρ=0.912, p<0.0001 |
| Reader TH | 1307±1557  [0-4260] | 11.2±5.4  [1-20] | 760±351  [110-1342] | ρ=0.723, p<0.0001 | ρ=0.644, p=0.002 | ρ=0.890, p<0.0001 |
| Reader HK | 474±515  [0-1801] | 10.5±7.2  [0-24] | 546±358  [0-1340] | ρ=0.801, p<0.0001 | ρ=0.726, p<0.0001 | ρ=0.907, p<0.0001 |
